# Protein Design with Deep Learning

**DOI:** 10.3390/ijms222111741

**Published:** 2021-10-29

**Authors:** Marianne Defresne, Sophie Barbe, Thomas Schiex

**Affiliations:** 1 Toulouse Biotechnology Institute, Université de Toulouse, CNRS, INRAE, INSA, ANITI, 31077 Toulouse, France; marianne.defresne@insa-toulouse.fr (M.D.); sophie.barbe@insa-toulouse.fr (S.B.); 2 Université Fédérale de Toulouse, ANITI, INRAE, UR 875, 31326 Toulouse, France

**Keywords:** computational protein design, artificial neural network, protein structure, inverse folding problem, language models, deep learning, generative models

## Abstract

Computational Protein Design (CPD) has produced impressive results for engineering new proteins, resulting in a wide variety of applications. In the past few years, various efforts have aimed at replacing or improving existing design methods using Deep Learning technology to leverage the amount of publicly available protein data. Deep Learning (DL) is a very powerful tool to extract patterns from raw data, provided that data are formatted as mathematical objects and the architecture processing them is well suited to the targeted problem. In the case of protein data, specific representations are needed for both the amino acid sequence and the protein structure in order to capture respectively 1D and 3D information. As no consensus has been reached about the most suitable representations, this review describes the representations used so far, discusses their strengths and weaknesses, and details their associated DL architecture for design and related tasks.

## 1. Introduction

The wide variety of natural proteins fulfills many different functions, from catalysis to specific recognition, transport, or regulation. This functional diversity makes protein crucially useful objects in a variety of settings, with direct applications in areas such as green chemistry, health, and biotechnologies [1].

The aim of Computational Protein Design (CPD) is to design proteins with new or enhanced properties (such as thermostability, binding affinity, etc.) or function (including ligand specificity and new activities) [2,3]. The most usual approach to CPD consists in choosing or *de novo* constructing a target backbone structure that could carry the function of interest and then identify a sequence that will fold onto this backbone and present the expected properties. In this case, the input of the problem is the target backbone with the targeted properties and the output is the designed sequence. This approach is sometimes referred as the *inverse folding problem*.

Importantly, the aim of protein design is not only to predict a sequence folding onto a target backbone, but also to bestow the design a specific function and required properties. Designing a useful new protein requires imposing specific global and local biochemical and geometrical constraints to dictate the desired properties. As an example, tight binding may benefit from the shape complementarity of the chosen fold to its target, but poorly placed charges, polar, or hydrophobic regions may prevent binding (see in [4] as an example). On the path from sequence to function, structure looks therefore as a crucial intermediary, but Machine Learning also offers pure sequence-based approaches that learn how to design sequences having a specific function, starting from a set of sequences carrying this function [5].

Based on its tremendous success in processing image, video, speech, and audio data [6], Deep Learning (DL) has been applied to many fields, including structural biology. In this area, the most visible success of DL has been obtained by AlphaFold2 [7,8] for protein structure prediction, demonstrating neural networks’ ability to learn a significant fraction of the complex relationships between sequence and structure in natural proteins. Deep neural networks (the type of architecture used in DL) being universal approximators [9], they are theoretically able to learn any function from enough data and computational power. Given the large amount of protein sequence and structure publicly available [10], this makes DL a very appealing approach to enhance protein design methods. However, applying DL to CPD is not straightforward. First, DL methods require suitably formatted input and output data. The chosen input representation must be adapted to protein of variable lengths and be able to concisely encode the relational information of the protein structure. Indeed, protein structures are naturally insensitive to translations and rotations; locally, only the relative orientation and position of structural motifs matters [11]. Ideally, the protein structure representation should account for these properties to make training more efficient. For now, there is no final consensus about which representation is best to capture protein structures and which neural architecture should be privileged.

In this review, after some background on CPD and Deep Learning, we present the different types of representation that have been used to represent protein data, both sequences and structures, when used for design or related tasks. We discuss their strengths and weaknesses, and detail the neural architecture used to process them.

## 2. Background on Computational Protein Design

### 2.1. Foundations and Methods

Computational Protein Design is becoming an increasingly attractive pathway to engineer new proteins with new functions or improved properties [12]. The first important challenge of CPD is the sheer combinatorial size of the sequence space: for a sequence of *n* amino acids, *n* choices among twenty amino acids, or more, have to be made, leading to $>20^{n}$ possible sequences, a number that quickly becomes larger than the number of atoms in the known universe.

The usual approach to design protein sequences relies on a target structure that is expected to carry the targeted function. This allows to formulate the design problem as an optimization problem: given a input backbone, find a sequence that maximally stabilizes the input backbone (and also fulfill the desired function) by minimizing a score function that usually combines the free energy of the resulting protein with other function-related criteria. This formulation is convenient to develop algorithms, but it should be noted that it makes CPD an ill-posed problem: the sequence is optimized for the target structure, but this structure may not be optimal for the sequence which ultimately may fold in a different structure [13,14]. Because of the many degrees of freedom of proteins and the complexity of free energy computations, the resulting problem remains intractable and simplifying assumptions have to be made [2,15]: the target backbone is assumed to be rigid, side chain flexibility is captured by a discrete collection of conformations (or rotamers) [16], and energy reduced to a very simplified form. Even with these simplifications, CPD remains a non-deterministic polynomial-time hard (NP-hard) problem [17]. For the sizes of the problem that need to be solved, stochastic and deterministic approaches have however often been able to produce working solutions. The most usual algorithm is based on simulated annealing, as implemented in the software Rosetta [18]. As an iterative algorithm, its computational cost is fully adjustable and solutions of good quality are often produced in reasonable time, but convergence cannot be reliably detected. Instead, provable methods exists that can always produce a solution of good quality, but their running time may grow quickly with the size of the problem. Historically, the first provable method was based on dead-end elimination and the A* algorithm, as implemented in OSPREY [2,19]. More recently, an AI-Automated reasoning solver, Toulbar2 [20], was able to produce proofs of optimality fast enough to point out Rosetta’s algorithm inability to always converge to an optimal solution [21].

The excitement that has been generated by the progress of Deep Learning has led to the proposal of DL-based tools targeted at various protein-design related tasks. Note that most existing structure-based DL-based tools target the creation of an amino acid sequence that will fold into a target rigid backbone, often without any explicit representation of a specifically sought function. These tools either directly produce a simple joint distribution over sequences (e.g., described through a Position Specific Score Matrix (PSSM)) or may predict one extra amino acid given some of its context (in terms of sequence and/or structure). The notion of protein function is instead often central in pure sequence-based DL design tools. They do not need a target backbone, but may instead start from a large multiple sequence alignment of proteins, sharing the targeted function.

**Figure 1:**
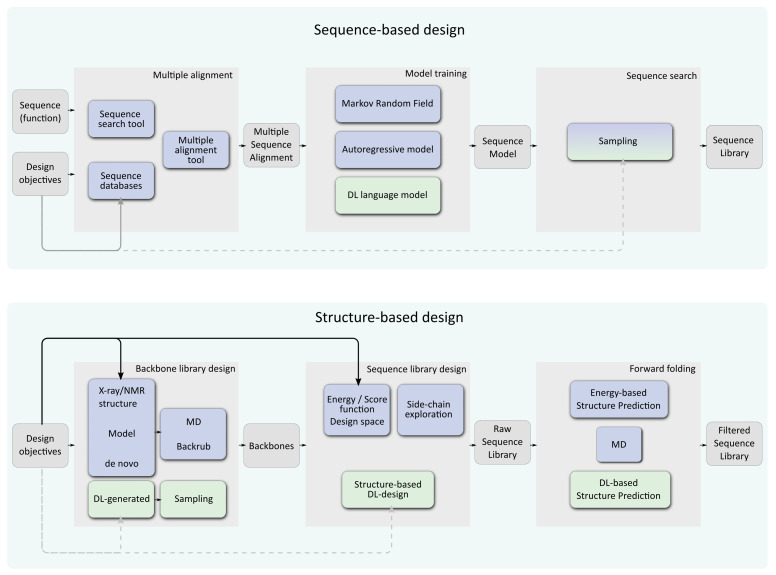
General workflows for computational protein design. Deep Learning-based approaches are represented in green, other approaches are in blue. The top workflow represents pure sequence-based approaches where a sequence, capturing a function is used to scan sequence databases in order to build a multiple sequence alignment. This alignment is used to train a machine or deep learning model that can be sampled to produce a sequence library. The bottom workflow is for structure-based computational protein design. One or several backbones (if multistate/flexibility is sought) are generated and possibly locally or globally perturbed. These can be exploited by usual energy/score function based method exploring a design space of possible rotamers (blue) or DL-based design (green). The resulting sequence library can be filtered using “forward folding”: randomized predictions are used to check if the sequence is predicted to reliably fold on its target backbone. The design objectives (beyond reaching the target fold itself) can be taken into account at different steps in these workflows (arrows). Traditional energy-based design offers several opportunities to inject these objectives in the design process: during backbone design, in the optimized score function, by combining them with the approximate free energy function used for optimization and also in the sequence/conformation space explored. In sequence-based design, such capabilities are strongly constrained by the landscape of existing proteins but some control may still be possible during the selection of the learning data-set and the sampling procedure. Most structure-based Deep learning approaches offer little or no control beyond backbone design (dotted lines).

Beyond pure sequence design, there are also additional tasks that the protein designer often needs to solve and for which DL provides new solutions (see Figure 1). Upstream of the design itself, structure-based methods require an appropriate input backbone. When an existing protein with no experimentally determined structure needs to be engineered, template-based modeling is used. This step will be greatly facilitated by the recent advance in DL-based structure prediction. Following the success of DL in the CASP13 competition (Critical Assessment of protein Structure Prediction) [7,22], many of the top methods integrated with success some DL components for CASP14 [23], including trRosetta [24], D-I-TASSER, and D-Quark [25]. Currently, top methods [8,26] can sometimes reach atomic accuracy on single chains, and can even deal with multimers [27]. For more information on DL for protein structure prediction, the reader is referred to the recent reviews in [28,29].

Other DL methods have been proposed that aim at generating backbones coordinates by learning how to generate distance maps [30,31,32], how to complete partial contact maps [33], or by directly generating 3D coordinates [34]. This may be useful for ab initio design and loop modeling. While some of these tools allow for sampling protein structures from a sequence or a sequence family, they often lack the ability to guide the generation process by a design target. Recent proposals however do introduce some preliminary form of control, using dedicated architectures [32,34] or loss functions [35]).

Following the design itself, it is usual to filter designed sequences to keep only those that are reliably predicted to fold in the target structure, a procedure called “forward folding”. Again, the recent advances in DL-based structure prediction tools will make this step more efficient and more reliable. We list these various tasks in Table 1. In this paper, we focus on the pure sequence design task, aiming at producing a sequence that should either fold in a target backbone or, for some, present a desired function.

### 2.2. Evaluation of Design Methods

Protein design methods need to be assessed, and there is no perfect metric. The most usual metric, native sequence recovery rate (NSR), evaluates whether design produces sequences similar to the native protein sequence of a natural given structure. However, with the restricted number of observed folds [61], it is known that many sequences will adopt a similar structure (degeneracy). Thus, a designed sequence with an NSR between 30 and 40% is already considered as satisfying, matching the sequence identity between homologous proteins [62]. It is also often impossible to directly compare the NSR of different methods if they have not been measured (and trained, when Machine or Deep Learning is used) on the same sets of target backbones [63]: some backbones are more constrained than others and this influences the likelihood of reconstructing the native sequence. The composition of the set of structures used for measuring NSR may therefore strongly influence the final numerical estimation. A much better evaluation consists in experimentally testing that the actual fold matches the targeted one, but this is an expensive process and few papers DL papers experimentally characterize the structure adopted by designed sequences (by X-ray crystallography [56] or circular dichroism [49]).

One should also not forget that the true purpose of most designs is not just to reach a suitable structure. Usually, the design target includes specific properties that are not captured by the backbone structure alone. This includes affinity, catalytic activity, ligand specificity, and ease of expression that needs specific essays. In practice, to have real added value compared to natural proteins, the designed proteins are often required to be functional in *non-natural* conditions in terms of temperature, pH, solvent, or ligand specificity, for example. In this situation, recovering the native sequence becomes increasingly unlikely and NSR, increasingly irrelevant.

## 3. Background on Deep Learning

In this section, we detail the notions of Deep Learning that are important to understand the protein design approaches described. Deep Learning, as a specific variety of Machine learning, is a method that tries to extract patterns from data [6]. Compared to other Machine Learning approaches, DL is highly efficient at extracting features from raw data, thus bypassing the complex task of hand-crafting informative features. It has shown unprecedented accuracy on various complex learning tasks. DL relies on a specific data structure, called an artificial neural network (see Section 3.2). A non-naive but simple example of it is the Multi-Layer Perceptron (MLP): it takes as input a vector of real numbers and processes it through a succession of linear transforms (defined by multiplicative weights, or parameters) alternated with nonlinear functions such as max(0,x), called *activation functions*. The final output, usually a small vector of real numbers, is then used to produce an answer to the targeted task. The general architecture (number of layers, sizes, activation functions) and the parameters need to be adjusted (trained) so that the output reliably produces correct answers to the targeted task, and not just on examples that were used for training (generalization, see Section 3.1). In supervised learning, this parameter tuning is achieved using a large collection of input vectors with associated known solution (or label) by minimizing a dedicated *loss function* using some variant of gradient descent. Efficient computations can only be reached using a variable number of Graphics Processing Units (GPUs).

### 3.1. Training

The exclusive data type that artificial neural networks deal with are tensors of real numbers, or multidimensional tables. It includes vectors, matrices, and beyond. The training data needs therefore to be first transformed into tensors to be processed by the network. The final output of the network, on its last layer, is then assessed by a numerical loss function that quantifies how well the learning objective is attained, empirically, using known answers (supervised learning) or not (unsupervised learning). Starting from the gradient of this loss, gradients on the neural network’s weights can be computed using a method called back-propagation and the weights updated using a simple gradient-based update.

The output of a neural network is also a tensor that can be interpreted in many different ways. In classification (predicting a class among *k* possible ones), the output is a vector of size *k* and the index of maximum value gives the predicted class. In regression (predicting a continuous number), a single number is produced. For each task, a specific loss needs to be used. For instance, given a protein sequence, one can ask the network to predict whether it is intrinsically disordered or not (classification), or to estimate its stability (regression). Training is usually supervised, comparing the predicted label to the true label and back-propagating the errors.

**Figure 2:**
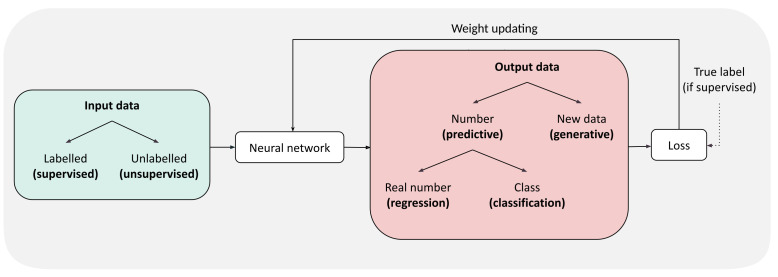
General pipeline of neural network training. Input data is in green, output in red. The types of model are written in bold.

Generative models have a more complex output because their aim is to generate new data (sample the probability distribution from which the training data comes). In the rest of the review, we will describe the two main examples of such models (see GAN and VAE in Section 3.2.4) that directly design new sequences (or structures). Training is often unsupervised as the data is just a collection of objects with no associated labels. These models therefore optimize specifically-crafted loss functions (sometimes loss functions that are learned by another neural network). Different types of models are summarized in Figure 2.

More complex situations may benefit from *transfer learning*, that may exploit both labeled and unlabeled data. We later describe approaches that train a first network on unlabeled protein sequences, then use intermediary output to enhance a second network, trained in a supervised fashion to predict the properties of the sequence.

To detect and avoid overfitting (lack of generalization to unseen data), the performances of the neural network are assessed on a separate, independent test data-set. These performances are evaluated quantitatively thanks to metrics adapted to the output and to the objective. For a proper evaluation of the network performance, it is crucial for the composition of this test set to be representative of its final usage conditions.

### 3.2. Architectures

#### 3.2.1. Convolutional Neural Network

The first non-naive DL architecture, the MLP we already mentioned, is a feed-forward network: the process that produces the output from the input shows no cycle. This architecture alone is already suitable for many tasks.

**Figure 3:**
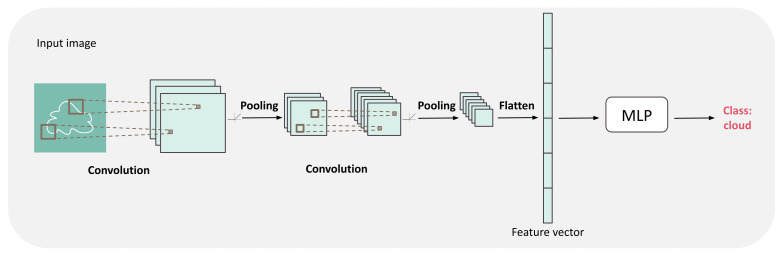
Convolutional neural network (CNN) architecture. The architecture is composed of a succession of convolutions, followed by an activation function and pooling layers to extract more and more global features from the image. Then, these features are flattened into a vector fed into a usual MLP which output is directly used to predict the class or regression value.

It has been refined to process images by restricting the linear transformations of MLP to local convolutions, an operation that computes a pixel state as a linear combination of neighboring pixels only. Through convolution, the output of a translated image is just translated. Convolutions are interleaved with *pooling* layers that merge blocks of (usually 2×2) pixels, thus reducing scale. The succession of convolutive and pooling layers, separated by activation functions, can extract more and more global features while taking into account neighboring information. Figure 3 summarizes the resulting Convolutional Neural Network (CNN). This architecture is of particular interest for protein design because the protein structure can be represented as a 3D image. Thus, all the CNNs developed to process images—a very dynamic field of artificial intelligence—are directly applicable to protein structures.

CNNs are an example of an architecture leveraging the symmetries of the problem (i.e., a translated motif stays the same motif) through translation invariance (translation of the input does not affect the prediction). Invariance can exist for all sorts of operations on the input, including rotation and permutation. An architecture that transforms its output in the same way as the input is said equivariant. Both invariance and equivariance are a major target in Deep Learning as they reduce the number of parameters to be learned without losing any power of representation. Fewer data are needed and performances improve in terms of training time and generalization ability.

CNNs have been improved to take into account multiple channels (used for color, with 3 RGB channels).

This has been directly used on protein structures (considered as a 3D image) by considering one channel per type of atoms (see Section 5.2). Empirically, CNN performance was observed to increase with the number of layers used (the depth), but this eventually lead to numerical issues during back-propagation, which slows down and possibly stops learning. To go deeper, residual connections [64] were introduced: the input of a layer is directly added to the output of following layers.

This idea has been pushed further in DenseNet [65], where each layer input is added to all residual blocks output and not just the next one. This is used in DenseCPD (see Section 5.2). Finally, if CNNs exploit translation-invariance, they are sensitive to rotation of the input. Thus, a rotated motif in the input—possibly a protein structure—will not be automatically recognized. A usual approach to tackle this issue is to use *data augmentation*: the training set is completed by similarly perturbed (rotated) images. However, this approach makes training longer and harder and using rotation-equivariant architectures is better.

#### 3.2.2. Recurrent Architectures

Text is a very common type of data that has been massively analyzed in Natural Language Processing (NLP), driving the development of dedicated architectures. From sentences, a succession of words, a very common task is next-word prediction: completing a partial sentence with an appropriate word. Here, training can be achieved from existing sentences, which are widely available. Protein sequences can be seen as sentences made of amino acid types, and thus can be processed by the same architectures.

**Figure 4:**
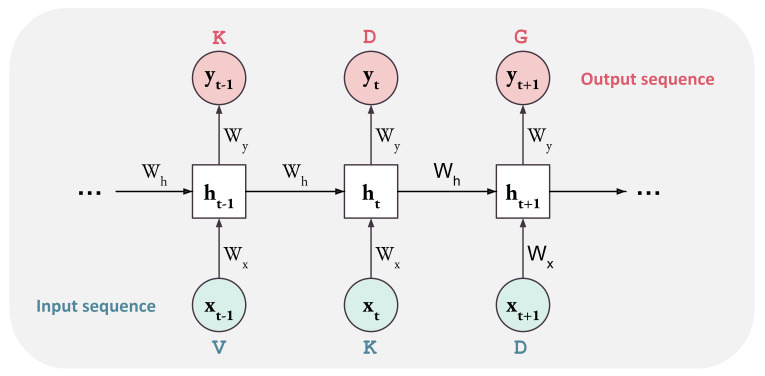
Pipeline of a Recurrent Neural Network. Elements *x* of the input sequence are processed individually by the same weights W.

Compared to images, that can always be scaled to a proper format, sentences have variable length. Recurrent Neural Networks (RNN) have been developed to process each word at a time by the same operation. The current output is computed from the current input word and the previous output, thus the name recurrent (see Figure 4). The idea, which is reminiscent of Hidden Markov Models, is to integrate the information from previous words to predict the next one. To preserve information from previous words, additional parameters, acting as a form of memory, have been introduced in LSTMs (long short-term memory) [66].

Individual words must be transformed in fixed size numerical tensors (vectors here). The most obvious approach is to sort all words and identify each by its position *i*. However, close numbers may correspond to totally unrelated and this makes training difficult. One-hot encoding represents word by a vector of 0s, except for a 1 at the *i*th position.

As explained later (in Section 4.2), such discrete data are often better represented using a *learned embedding*, where each word is associated to a specific real vector of a chosen fixed size. Embeddings can be learned by Languages Models (LMs) [67,68] to give them a semantic flavor: similar embeddings (in terms of cosine distance between vectors) should have a similar meaning.

Ultimately, they can even be used to get a working semantic “algebra” as often exemplified by the (approximately satisfied) equation (king − man + woman = queen) [69]. Suitable learned embeddings are critical for the performances of various models built on top of them. Various architectures have been suggested for LMs, and many of them have been applied to protein sequences to “learn the language of life” [39]. These approaches are presented in Section 4.2.

#### 3.2.3. Attention Models

A more recent approach to handle sequential data replaces recurrence by so-called *attention* mechanisms, popularized in the Transformer architecture [70]. Recurrent architectures have an inherent difficulty in exploiting long-distance interactions in a sequence [71]. Attention models have been precisely designed to fight this limitation by learning to identify which part of the input is important (and how) for the prediction. The neural net can then focus on those parts, no matter their range, to predict the output.

For instance, when predicting protein contacts, attention can make each residue attend to only few other residues, which are therefore more likely to truly be in contact [72].

To allow for considering any distant interaction, the size of the input sequence is bounded to a maximum and data processing is parallel instead of being sequential. This makes the process potentially more efficient but also more memory intensive, especially for large maximum lengths.

#### 3.2.4. Generative Models

**Figure 5:**
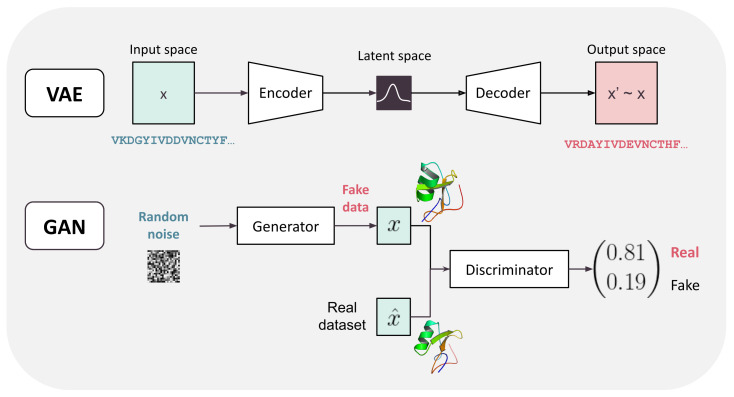
Examples of generative models. From top to bottom: Variational Autoencoder (VAE) and Generative Adversarial Network (GAN) models. The encoder, decoder, generator, and discriminator may be neural networks of any type (including MLPs, recurrent, and attention-based).

Generative models aim at learning the unknown distribution of the training data in order to generate new data from the same distribution. Figure 5 gives a graphical representation of the architecture of the two most usual DL-based generative models: autoencoders and Generative Adversarial Networks. DA simple generative model can be learned using autoencoders: an input *x* is successively reduced in dimensionality by an encoder, producing an internal low-dimensional representation of the input *x* (a latent representation). This representation is then decoded to produce back the original input *x*. This encoder–decoder pair is trained as a single network that must learn the identity function. This process has been simplified in Variational Autoencoders (VAE) [73] with a simpler latent distribution representation. The learned latent representation may be useful as a learned embedding of the input but also for generative purposes: new data can be generated by sampling the latent space and decoding it. However, this often leads to inconsistent output because the fraction of the latent space that describes correct output may be very small.

Another popular approach is Generative Adversarial Networks (GAN) [74]. GANs also use a combination of two neural nets: a generator that learns how to generate new data (from the same distribution as the training data) and a discriminator that learns to predict whether its input is out-of-distribution or not. The training objective, encapsulated inside the loss, encourages the generator to fool the discriminator and the discriminator to reject out-of-distribution data, thus the name adversarial. After training, the generator alone is used to generate new data. Training GANs can be challenging as both learners need to learn at comparable speed [75]. GAN and VAE are unsupervised methods but the inner architecture of their networks are of the same type as discussed before (MLP, CNN, attention-based), depending on the type of data to handle.

## 4. Representation of the Protein Sequence

**Figure 6:**
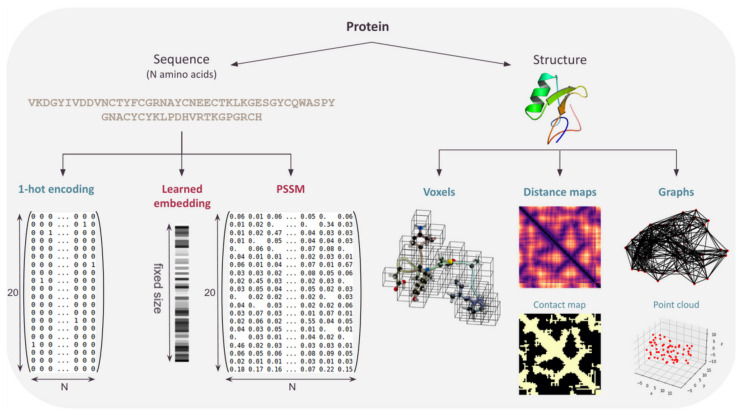
Various protein structure and sequence representations.

The CPD problem requires to produce sequences as output, and sequences are also often present in the input, possibly as the sole input in pure sequence-based approaches. In this case, compared to structure-based approaches, training data-sets can be extremely large: more than two-billion sequences have been clustered in the “Big Fantastic Database” [8]—a tiny fraction of them being associated with a reliable functional label (80,000 with Molecular Function Gene Ontology in SwissProt [76]). Given that neural nets only accept tensors for input and output, protein sequences must be formatted into a tensor. Three major approaches have been used here, as summarized in Figure 6.

### 4.1. One-Hot Encoding

As already described, a protein sequence of length *n* can be one-hot encoded. Each amino acid is represented by a Boolean vector of size 20 (or more if gaps/unknown amino acids need to be represented), all concatenated into a n×20 matrix representing the full sequence. This simple encoding is widely used in DL protein design methods based on language models (see Section 4.2) or in generative models that try to learn the distribution of the training set (sequences) to generate similar data (hopefully, sequences with the same property as the training set).

These approaches can be used to design new sequences that should carry the function of the input training set, usually an MSA built from one chosen functional protein, something that was already been proven to be feasible using generative probabilistic models such as Markov Random Fields [5,77,78] or autoregressive models [43], closely related to Bayesian networks [79]. While they explicitly target a function, these methods look intrinsically limited to sampling the distribution generated by nature, which is not ideal to design proteins offering new functions, or to extend the scope of an existing function to non-natural conditions.

Generative models such as GANs [42] have been used to design new functional sequences (here a malate dehydrogenase MSA was used as a starting point), VAE [46] to craft metal-binding site to sequences or predict sequences folding according to a general topological description, encoder/decoder architecture [47] to design peptide signal sequences. As all these approaches bypass the need of an intermediate structure and use a simple sequence representation, they are not the core of this review. For further reading, we refer the reader to recent reviews [80,81].

One-hot encoding is a limited representation that does not integrate much information per se. It makes all the configurations equidistant, whereas some sequences are biologically or physically closer than others. Thus, neural networks applied to such input cannot leverage possible crucial information that could be captured by learned embeddings.

### 4.2. Learned Embedding

A more informative input than a one-hot vector can be obtained through a suitable sequence embedding, a fixed-size vector representing the sequence, learned to capture important information in the context of the targeted task. Several NLP-inspired approaches tried to decipher “the language of life” [39] by considering the protein sequence as a sentence and the amino acids as words. A natural sequence corresponds to a meaningful and correct sentence. In this context, “next word prediction” approaches can be directly leveraged to predict the next amino acid or to recover a masked sequence [40].

This tight connection with NLP has attracted a lot of interest. In order of publication, NLP approaches have been adapted to protein word embeddings [36], recurrent architectures by UniRep [37], Embedding from Language Models [38] by SeqVec [39] and the attention-based Transformer [40]. Finally, ProtTrans [41] compared six successful NLP architectures on a dataset of an unprecedented size (2122 million proteins, 8 times larger than those previously used). They all produce a fixed-length embedding for each amino acids, then average them to obtain the sequence embedding.

One major drawback of protein embedding is the computational cost to learn them. The most complex of the models mentioned above, ProtTrans, was trained using Summit, the world’s second-fastest computer. Such costs are prohibitive for most research groups, meaning the learned protein embeddings can be impactful only if the trained model is publicly available, as it is the case for ProtTrans.

If the embeddings are meaningful by themselves (the embedding space is clustered along protein functional, biophysical and structural properties [40,41]), they are especially interesting when used as inputs for subsequent supervised tasks. Indeed, simple models on top of such embedding outperformed complex models taking one-hot encoded amino acids as inputs on prediction tasks [82].

These embeddings have been used for protein design: ProGen [83] framed the problem as “next amino acid prediction”, each of them being represented by a learned embedding. The output sequence is built iteratively by a Transformer architecture (see Section 3.2.3) from the partial sequence predicted so far and from conditioning and taxonomic tags describing the desired properties with keywords (such as cellular component, biological process, and function terms).

Another approach used UniRep embeddings for *in silico* directed evolution [48]. Embeddings were first fine-tuned on sequences related to the target protein. Then, a low number (24 or 96) of experimentally characterized mutants of the wild type were used to train a linear regression predicting the activity from the fine-tuned embeddings. Finally, top sequence candidates were selected by in silico-directed evolution based on the activity predicted by linear regression. About 10% of them displayed an activity higher than the wild type.

### 4.3. Position-Specific Scoring Matrices

Neural networks designed to predict sequences often chose to output a tensor which can be directly interpreted as a Position-Specific Scoring Matrix [84]. In most cases, these scores are transformed to a probability distribution over amino acids using a “softmax” function at the final layer [50,54,55,56,57,58]. Softmax combines exponentiation with normalization to transform arbitrary real vectors into vectors that can be interpreted as a multinoulli probability distribution. PSSM are also frequently used as a simple representation of sets of (aligned) input protein sequences [13,52,84]. A general limitation of PSSM is that they ignore interactions between different residues, which is physically untrue. Pairwise interactions, at least, seem crucial to capture some of a protein function [5].

This simple probabilistic-like output is very convenient however. Specific probabilistic measures such as cross-entropy can be directly used as losses. Moreover, this probability may encode the conditional probability of each amino acid, given the environment at each residue [13,46,50] to ultimately sample a diversity of sequences from the same network prediction, e.g., using a Monte Carlo algorithm [56]. This last approach reaches state-of-the art prediction accuracy for a single residue given its environment, approximately 57% [56]. This metric should not be confused with the native sequence recovery rate, which compare the whole designed sequence with the native sequence and which is usually between 30 and 40% depending on the test set [49,54,63,65].

Residue-level accuracy allows us to get some insight on the inner representations learned by the neural network. ProDCoNN approach, based on a CNN, found that their confusion matrix is similar to the usual BLOSUM62 [85] protein similarity matrix. It means the neural network confounds similar residues and has been able to capture the natural degeneracy of proteins. This was also observed by Alley et al. [37] who found a better performance on buried residues [56,57], which is consistent with the fact that these residues are more constrained.

## 5. Representing the Protein Structure

Protein design, when framed as the *inverse folding problem*, requires an input protein backbone. A protein structure being a complex object, the choice of its representation is both critical and non-trivial. The obvious insensitivity of proteins to translation and rotations should ideally be directly exploited in the associated architecture. The first approaches used sequential representation of the structure, which was convenient for use but did not leverage the geometric properties of protein structures. To date, there is still no consensus on what representation is the most adapted to learn from protein structures [53]. Three major types of representation are used: voxels, distance maps, and graphs (Figure 6). In this section, we describe the use, benefits, and drawbacks of each of these for CPD as well as the type of neural network used to process them (see Table 2).

### 5.1. Sequential and Hand-Crafted Representations

Given the input protein backbone, one straightforward way to represent it as a tensor is to consider it as a sequence of distances, angles and dihedrals (as usual in molecular modeling). While rotation- and translation-invariant, this 1D representation is not ideal in the context of Deep or Machine learning because of its sensitivity to noise through the well-known “lever effect”: a tiny change in one dihedral angle may translate in very large changes in distant Cartesian coordinates [50,55,86]. Instead, each position of the sequence can be associated with structural features (characteristics deemed to be significant for the learning problem), possibly completed with other features related for example to the target design properties. Such an approach was used in the first DL-based CPD systems, starting with SPIN [86] and its extension SPIN2 [55]. Both used sequence-local and non-local features including the above 1D representation: backbone torsion angles, interaction energies with side-chains, and sequence profiles obtained by comparing 5-residue fragments of the target structure to a template library, keeping sequences of structurally similar fragments. Non-local features included contact numbers and interaction energies between a residue and the rest of the backbone. All theses features were stacked and fed into a 3-layer MLP to produce protein sequences. Wang et al. [50] used a similar architecture on simple per-residue geometric and structural features (such as dihedral angles, $C_{\alpha }-C_{\alpha }$ distances or the solvent accessible surface area). A sliding window incorporated information from the *k*-nearest neighboring residues in 3D space. Both output a PSSM.

Instead of working at the residue level, Greener et al. [46] represent the input fold using a context-free grammar based on its secondary structure elements [46]. A flat tensor of the one-hot encoded rules of this grammar are fed into a VAE to produce a sequence likely to adopt the input general fold topology.

The main advantage of using hand-crafted structural features is to obtain a fixed-size feature vector, which is required by many DL architectures, including MLPs and CNNs. Even recurrent models, that accept sequences of variable length, need a fixed-size word embedding (the amino acid feature vector). The obvious drawback resides in the features themselves. Crafting and selecting suitable features, with enough information to learn the complex sequence/structure relationship, is a hard task, and important information may just be lacking (or some be redundant). This does not leverage the ability of neural network to automatically extract features which also requires to feed the neural net with an entire structure. Thus, other representations are needed.

### 5.2. Voxel Representation

As a three-dimensional object, a protein structure can be directly analyzed as volumetric data that can be fed into a 3D-CNN: the space is discretized into cubic voxels of typically 1 Å side. Atoms within each voxel are counted and a Gaussian filter is applied to the discrete count to produce an occupancy map. Each type of atom is counted independently and treated as different channels, specifically RGB channels [56,57,58,59]. All approaches consider nitrogen, oxygen, and carbon atoms, but some also represent sulfur and phosphorus [56], or differentiate $C_{\alpha }$ and $C_{\beta }$ from other carbons [57].

The usually considered task is to predict an amino acid given its local environment. The input of the network is a box of size about 20 Å, centered on the target residue. The geometrical environment is canonized to ensure rotational and translational invariance. This input, of shape C×(volume of the cube) with *C* the number of channels, is fed into a 3D CNN, which is trained on existing proteins, to output a probability over the 20 possible amino acids. The details of the architecture differ between approaches: DenseCPD stacked Dense blocks [65] while ProDCoNN used three parallel layers of different filter sizes to catch information about covalent bonds, bond angles, and dihedral angles [58]. This task is also useful for single-point mutation prediction [59], or for protein design, either indirectly (using the prediction to reduce the search space of Rosetta [57]) or directly by taking the maximum probability for each amino acid [58], or by sampling [56]. While DenseCPD outperformed several competitors in a recent benchmark experiment [63], these results have to be taken with caution because the training and testing data-sets used were not proven to be separated in the evaluation.

The main interest of this approach is the ability of 3D-CNNs to take into account the geometry of the protein structure. 3D-CNNs are used to identify structural motifs, independently of their scale or position, which is critical to decipher the sequence/structure relationship. The sensitivity of CNNs to rotation is canceled by the use of canonized frames but this may not be always sufficient in the context of protein complexes [87]. This limitation can be bypassed by rotational data-augmentation, which increases computation time. Discretization also requires to settle compromises between computational complexities and fidelity.

### 5.3. Distance Maps

A distance map is a 2D representation of a protein structure. It is a n×n matrix (*n* being the length of the protein) giving the distance between $C_{\alpha }$ atoms of each pair of amino acids. Contact maps are binary maps obtained by putting a distance threshold on the distance map. Both contact and distance maps have been massively used for protein structure prediction methods, including AlphaFold [7], trRosetta [88], and their successors [8,26].

These structure prediction networks can be partially inverted using so-called symbolic gradients: when an input sequence and an output structure are given, backpropagation can not only compute gradients on the weights (for training), but also on the input. Norn et al. [13] relied on trRosetta: a CNN with residual connections (see Section 3.2.1) predicting a distance map (and more) from a sequence. A random sequence is fed into trRosetta, and the differences between the predicted and targeted distance maps are back-propagated to the input sequence (or more precisely, its encoding). Iterated, this process tends to optimize the input sequence so that it folds in the target structure. The major claimed advantage here is that the resulting sequence seems to avoid the pitfall of the usual ill-posed CPD problem: the predicted sequences seems to implicitly avoid the existence of alternate stable backbones. Another method exploited trRosetta to hallucinate ideal proteins [44]. Starting with a random sequence, a single random mutation is introduced and its distance map computed by trRosetta. The mutation is kept with the objective of encouraging the sequence to be different from background and the process iterated. The resulting proteins have nature-like structure and a low sequence similarity (10%) with known proteins. Twenty-seven sequences, out of 129 (20.9%), were experimentally shown to produce folds consistent with the predicted structure. The two approaches have been later combined for local hallucination [35].

Distance map can also be used directly to represent an input structure. SPROF extended SPIN2 by incorporating 3D information in the form of a contact map [54]. They processed the map as an image, and the corresponding sequence as a caption. Then, taking inspiration from the usual “image captioning” task, they coupled a RNN and a CNN to output a PSSM, from which a designed sequence is produced.

As a 2D representation of a 3D structure, contact and distance maps offer several advantages. They are a low-dimensional, which makes computations efficient. They are images that benefit from all DL methods developed in the field such as CNNs with residual connections [7,54,88]. Finally, they are invariant for rotation or translation of the protein structure. The dimension reduction leads to the loss of some geometric information that can often be recovered [89].

### 5.4. Graph Representations

#### 5.4.1. Graphs

Graphs are well-suited to represent relationships, here between residues in a protein structure. In the most basic graph, each node, or vertex, corresponds to one residue, and edges connect pairs of residues within a distance threshold. Such a graph is equivalent to a contact map. A graph can be advantageous when there are few interactions between amino acid (modeled by a small distance threshold). With a sparse graph (with few edges compared to a complete graph), computations can be more efficient than on a distance map which explicitly represents all pairwise interactions. Contact maps are naturally sparse as the number of contacts of each residue is bounded. Moreover, nodes and edges usually can contain more attributes than just the amino acid type and distance respectively. As additional edge attributes, Strokach et al. [49] used the number of residues in between the nodes in the sequence. Ingraham et al. [51] consider also direction and orientation from local coordinate systems, as well as dihedral for extra node attributes.

Such annotated graphs can be processed by a dedicated Graph Neural Network (GNN) architecture. If recurrent neural nets can be seen as generalized Hidden Markov Models, GNNs are inspired by graphical models message passing algorithms [90]: each node sends information to its neighboring nodes, then each node aggregates the information received from its neighbors to update its state. The first step is done by defining a graph convolution, an operation that generalizes the usual convolution for graph [91]. The difficulty here is that a node can have a variable number of neighbors, so graph convolutions must operate on a variable size input. Most approaches define their own graph operations, including ProteinSolver [49]. Starting with a target structure and a masked sequence to complete, node and edge embedding are produced through several graph convolution and aggregation blocks. The node embeddings are then fed into feed-forward layers to predict the missing residues. ProteinSolver was ranked as one of the worst performers in a recent evaluation [63], but GNNs offer a lot of hyperparameters to tune.

Graphs can also be processed by an adapted Transformer architecture. Ingraham et al. [51] used an encoder with three attention layers to produce node embeddings. Then, an autoregressive decoder produces the output sequence, residue after residue.

Graphs are of course very convenient to capture spatial neighbors information, but they lack the ability to capture fine grain geometry as CNNs on voxels. In order to benefit from the advantages of both approaches, the Geometric Vector Perceptron approach defines a specific graph convolution that is rotation and translation equivariant [53] as convolution for translation. The resulting operations are also computationally more tractable than other equivariant approaches [92]. With their architecture, the output sequences achieved state-of-the-art results in terms of native sequence recovery rate (40.2%, see Section 2.2 for caution on interpreting such numbers).

#### 5.4.2. Point Clouds (3D Coordinates)

The most brutal way to represent a structure is probably as a point cloud, the list of all 3D coordinates of its constituents, much like a PDB file. This dense information can be filtered to just keep the coordinates of $C_{\alpha }$ carbon atoms [52], or an all (heavy) atom representation can be preserved [60].

These points can then be used as nodes in a graph, and thus they can be processed by a GNN-like architecture such as MimNet [52]. When fed with a PSSM, MimNet returns protein structure 3D coordinates. It can also be reversed to predict a PSSM from coordinates. Thus, MimNet is able to do both folding (forward) and design (background). The reversibility of the architecture is achieved thanks to a specific graph convolution, inspired by a differential equation describing molecular dynamics. MimNet is simultaneously trained on the two tasks so that the design is improved when the structure is better predicted.

Coordinates can also be considered as a set of points, as in Atom Transformer, a network learning an energy function to predict the protein conformation. As its name suggests, it is based on a Transformer architecture that processes a set of atom coordinates and features (atom type, amino acid type, and position in the side chain). Atom Transformer is trained in an unsupervised fashion, by maximizing the likelihood of the learned energy function [60]. The conformation with the lowest energy is predicted to be the native conformation. In this architecture, the attention mechanism replaces the graph convolution for the task of seeking information from the neighbors.

Point clouds can be directly processed by rotation-equivariant operations, avoiding data augmentation. However, these operations are costly in terms of computation time [92]. By comparison with the general graph representation described in the previous Section 5.4.1, point clouds do not reduce the geometric information about the structure.

## 6. Conclusions

In the last couple of years, Deep Learning has been widely used to propose new protein design approaches, either by directly generating a sequence from a set of sequences carrying a function or by using a backbone target to predict a sequence likely to fold onto it. In either case, sequences and structures need to be represented as tensors. As the literature shows, there is a wide variety of choices and no clear consensus on the preferable representations or architectures for design.

When compared with classical CPD methods, pure Deep Learning models can often avoid the restricting assumptions that are present in the usual *energy-based inverse folding* formulation [13] of CPD. These simplifying assumptions may reduce the size of the search space to make computations less intractable by targeting a fixed chosen backbone. This is something that pure sequence based DL (or ML) approaches do not need to do. When structure is explicitly considered, alternative backbone conformations seems to be also implicitly accounted for in reversed structure prediction based methods for design [13], avoiding the ill-posed nature of the inverse folding formulation. Finally, DL-based approaches do not need discretized side chain geometry, and some of them explicitly model flexible backbones [51], something that is far from simple for energy-based methods [14]. However, except for a few of them (see Section 2.2), these methods have not been experimentally validated.

Beyond this, most of the existing structure-based proposals are focused on designing sequences for a fold. In practice, various additional constraints need to be imposed on the chemical composition (sequence), geometry, or stability of various critical regions. Practical designs rarely require producing a new sequence for a known fold. Pure sequence-based approaches starting from a known family sharing a function do target a function, but may be limited to sampling the distribution of natural proteins. Instead, computational protein design is most useful when radically new functions or properties need to be created.

Therefore, one of the main weaknesses of DL and ML approaches to protein design probably lies in the difficulty they have to produce specifically targeted out-of-distribution protein sequences which would fold and work in non-natural conditions in terms of pH, temperature, ligand-specificity, target, catalytic activity, or other enhanced properties. This is often dealt with, in energy-based design, using multiple criteria or constraints capturing not only energy, but also design targets. Some DL approaches have tried to incorporate constraints to their model by adding additional features, such as as supplementary one-hot vector indicating the desired type of metallo-binding site to add [46]. Imposing constraints on DL models output is a challenging problem for Deep Learning in general, for which specific losses have been proposed [93]. This usually requires a new training every time a new constraint needs to be enforced.

Recent advances in protein structure prediction, which can produce predictions close to atomic resolution [8], will have consequences in protein design. When used for forward folding, DL-based structure prediction should enable a more precise assessment of predicted sequences. Additionally, neural networks can also be reversed to guide design [94], as previously done with trRosetta [13]. By adapting the loss function to the design objective, authors designed *de novo* monomers, protein complexes, and oligomers able to switch conformation. However, this method shares the limitations of all DL-based methods on the enforcing of additional constraints on the predicted sequence. Our own prediction is that energy-based and DL/ML-based methods still have a long way to go and need to learn from each other. Effective design requires approaches that can combine the specific strengths of each approach and that are also experimentally validated.

## Figures and Tables

**Table 1:** Protein design-related tasks tackled by DL.

Task	Approach
Learning sequence embedding	[36,37,38,39,40,41]
Prediction of sequences from other sequences	
*from sequences of the same family*	[42,43]
*from a random sequence*	[44,45]
*from any sequences*	[46,47,48]
Backbone generation	[8,26,31,32,33,34,35]
Prediction of sequences from input structure	
*by reconstructing the native sequence*	[49,50,51,52,53,54,55]
*by predicting residues given their local environment*	[56,57,58]
*by inverting a folding network*	[13]
Prediction of single-point mutation	[43,59]
Prediction of side chain orientation	[60]
Forward folding	[8,26]

**Table 2:** Explicit representations of the structure and the neural architecture they are fed into.

	Hand-Crafted	Voxels	Distance Map	Graph	Point Clouds
MLP	[50,55,86]	-	-	-	-
CNN	-	[56,57,58,59]	[13,44,54]	-	-
GNN	-	-	-	[49,53]	[52]
Transformer	-	-	-	[51]	[60]
Generative	[46]	-	[45]	-	-

## Data Availability

Not applicable.

## Abbreviations

The following abbreviations are used in this manuscript:

CNN	Convolutional Neural Network
CPD	Computational Protein Design
DL	Deep Learning
GAN	Generative Adversarial Network
GNN	Graph Neural Network
MLP	Multi-Layer Perceptron
MSA	Multiple Sequence Alignment
NLP	Natural Language Processing
PDB	Protein Data Bank
PSSM	Position-Specific Scoring Matrix
RNN	Recurrent Neural Network
VAE	Variational Autoencoder
